# Research on a capacitive particle analysis smoke detector

**DOI:** 10.1038/s41598-024-62400-9

**Published:** 2024-05-17

**Authors:** Boqiang Wang, Xuezeng Zhao, Yiyong Zhang, Zigang Song, Zhuogang Wang

**Affiliations:** 1 Harbin Institute of Technology, 92 Xidazhi Street, Harbin, 150006 Heilongjiang China; 2 China State Shipbuilding Corporation Limited 703 Research Institute, 35 Honghu Street, Harbin, 150010 Heilongjiang China

**Keywords:** Extreme early fire detection, Smoke concentration detection, Capacitive detection, Multiscale signal processing, Electrical and electronic engineering, Mechanical engineering

## Abstract

Smoke detectors face the challenges of increasing accuracy, sensitivity, and high reliability in complex use environments to ensure the timeliness, accuracy, and reliability of very early fire detection. The improvement and innovation of the principle and algorithm for smoke particle concentration detection provide opportunities for improving the performance of the detector. This study represents a new refinement of the smoke concentration detection principle based on capacitive detection of cell structures, and detection signals are processed by a multiscale smoke particle concentration detection algorithm to calculate smoke concentration. Through experiments, it was found that the detector provides effective detection of smoke particle concentrations ranging from 0 to 10% obs/m; moreover, when the detection accuracy is greater than a certain number of parts per million (PPM), the sensitivity of the detector can reach the PPM level; furthermore, the detector can detect smoke particle concentrations higher than the PPM level accuracy even in an environment with a certain concentration of petroliferous and dust particles of different sizes.

## Introduction

Very low concentrations of smoke particles can be effectively detected during very early fire detection. This approach of capacitive particle detection method can effectively control the further development of fires and minimize losses of all kinds of fires. Unfortunately, there are greater than 100,000 cases of no alarm generation or alarm failure^1^, and more than 200,000 false alarms were responded to by fire departments^2^. These factors are expected to result in unnecessary losses, waste of firefighting resources, and declining public confidence. The fast and accurate detection of smoke particles from very quick fires is critical for avoiding losses and saving lives. It is required that the detector has a sensitivity of PPM level, and a smoke concentration measurement range of 0–20% obs/m, and power consumption should be kept under 30W.

Smoke concentration detection technology confronts the challenges of responding to the effects of interfering particles in complex environments, false alarm resistance, and adaptation. The interfering particles mainly come from airborne dust particles, oil and gas particles, and water vapor particles. The concentration of interfering particles usually ranges from 0.05 to 5%obs/m. Conventional point smoke detectors are unable to cope with harsh and intrusive environments^3^. Photoelectric smoke detectors are unable to distinguish between particle signals of different sizes, but the detector response speed increases when the emitting light source is a green LED^4^. Very low-concentration smoke particles (0.1%obs/m) released from very early fires can be effectively recognized by a photoelectric aspirating smoke detector, and this type of detector has achieved successful commercial application^5^. However, this approach can only partially eliminate the effect of other interfering particles through the filter and cannot distinguish the particle type. These factors significantly limit the applicability of the detector: the impact of the airflow direction on the mounting angle of the detector needs to be considered when designing the layout style of the pipeline^6^; the air sample pipeline needs to be complexly modeled in 3D to verify the reasonableness of the pipeline layout^7^; and the trajectories of smoke particles need to be identified by using computational fluid dynamics^8^. The false alarm resistance of a detector can be improved by adding a combustible gas detection module for alarm calibration^9^. A capacitive bending smoke sensor can increase its sensitivity by increasing the component contract area. However, it is still not able to distinguish between the types of particles, and false alarms can still occur^10^. A capacitive smoke sensor based on MEMS technology can detect smoke generated by hydrogen-containing substances during the smoldering stage. But, it is not sensitive to smoke particles by combustion of carbon-containing substances^11^. However, this approach also affects the sensitivity of the detector to a certain extent. While very low concentrations of smoke particles generated by very early fires are effectively detected, the effective identification of particle types is still a problem. Moreover, the false alarm rate of the detector will increase, and its reliability will be greatly affected in complex environments where oil gas particles and dust particles of different sizes are present. The diameter of smoke particles usually ranges from 0.1 to 100 μm.

In this study, a structure for analyzing and detecting smoke particles based on capacitive detection element cells is designed, and particles of different sizes will form mixed signals with different amplitudes and frequencies when they pass through the detection structure. A multiscale algorithm is used to detect smoke particle concentrations by sequentially analyzing mixed signals via time–frequency domain analysis, extracting smoke particle signals, sensitizing smoke signals, and calculating smoke concentrations. On the one hand, the detector will have higher detection accuracy and sensitivity because smoke particles are detected by the newly designed capacitive detection cell. On the other hand, the detector can differentiate signal characteristics effectively between different particles through the newly designed particle detection structure and algorithm so that the reliability of the detector increases in complex environments. The sensitivity, accuracy, and reliability of the proposed method were verified through a limit concentration detection experiment, smoke concentration detection experiment, and anti-interference ability experiment, respectively.

## Capacitive smoke particle detection principle and design

### Capacitive particle analyzer detector structure

**Figure 1:**
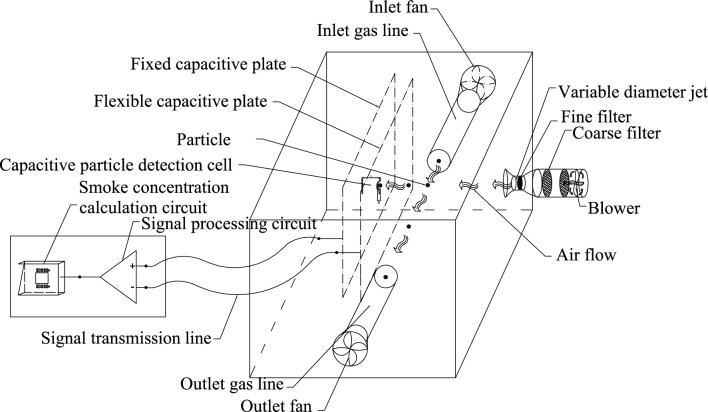
Capacitive particle analysis structure schematic.

As shown in Fig. 1, the capacitive particle analysis structure mainly consists of a pair of capacitive particle detection plates, a gas sample sampling path, a motive air path, and a signal processing circuit. Capacitive particle detection plates consist of a fixed capacitive plate and a flexible capacitive plate for detecting the particle type. The gas sample sampling path consisted of inlet/outlet fans, an inlet/outlet gas line, and a particle detection chamber to sample the air samples. The power gas path consists of filters, a blower, and a variable diameter jet exhaust to provide the kinetic energy for the sampled air sample to collide with the flexible capacitive plate.

### Particle detection principle

**Figure 2:**
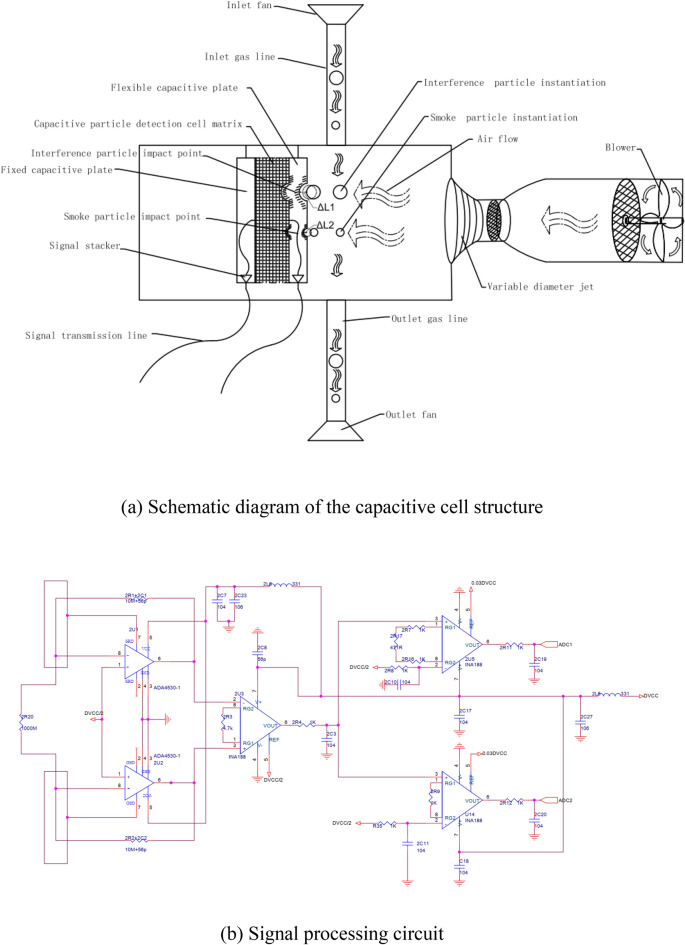
Particle detection schematic and signal processing circuit.

As shown in Fig. 2a, The signal stacker is used to accumulate the signals measured by all capacitance cells. The schematic diagram of the interface circuit is shown in Fig. 2b. smoke particles and interference particles are simultaneously inhaled into the particle detection chamber by the inlet fan. The air inhaled by the blower will be purified into clean power gas after passing through two layers of coarse and fine filters. Inhaled smoke particles and interference particles are blown by such gas to the flexible capacitive plate and collide with it. Suppose that vertical deformations of ${\Delta L}_{1}$ and ${\Delta L}_{2}$ are formed by a collision between interference particles and smoke particles on the flexible capacitive plate, respectively. Then, the capacitance on the capacitance cell changes as follows:

1 $$\left\{ {\begin{array}{*{20}c} {C_{{\vartriangle L_{1} }} = \frac{\varepsilon \cdot A}{{d - \vartriangle L_{1} }}} \\ {C_{{\vartriangle L_{2} }} = \frac{\varepsilon \cdot A}{{d - \vartriangle L_{2} }}} \\ \end{array} } \right.$$

where $C_{{\vartriangle L_{1} }}$ and $C_{{\vartriangle L_{2} }}$ are the capacitance variations generated on the impinged capacitance cell by interference particles and smoke particles, respectively; $d$ is the distance between the fixed capacitive plate and the flexible capacitive plate before the collision; and $\varepsilon$ is the permittivity of the capacitor, $A$ the relative projected area of the two capacitive plates.

A fixed DC voltage $U$ is applied between the fixed capacitive plate and the flexible capacitive plate. A precision sampling resistor is connected in series between two signal stackers of the fixed capacitive plate and flexible capacitive plate, and the signal stacker is used to collect the electrical signal produced by capacitive cells. Induced currents flow through the sampling resistor, and a voltage is produced when the change in capacitance is caused by particle impacts on the flexible plate.

### Capacitor detection cell design

The particles will only collide with the flexible plate in the normal direction. Because, the particle is only acted upon by the force in the normal direction. The normal force comes from the blower.

**Figure 3:**
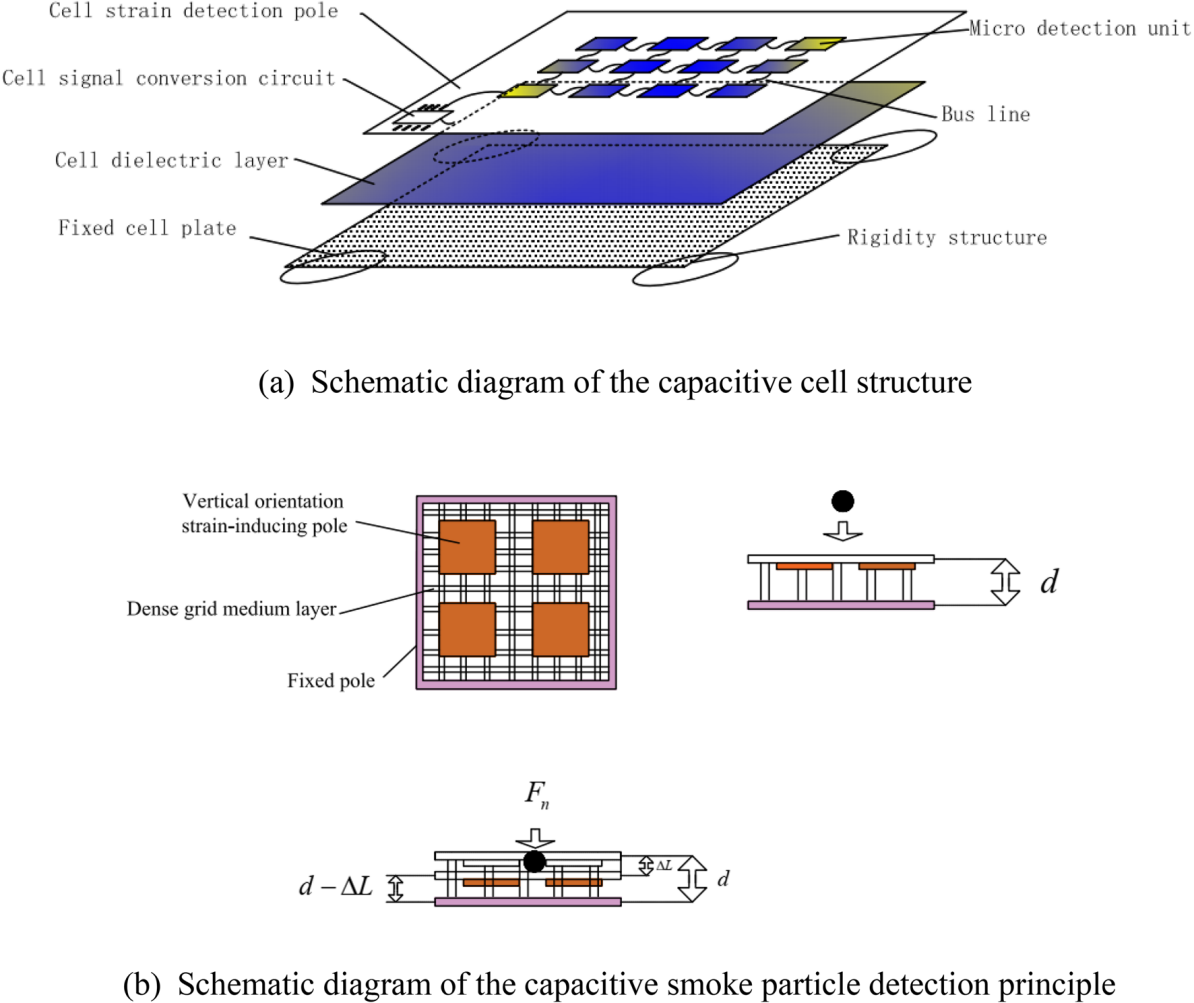
Capacitive detection cell.

The vertical orientation of the capacitive cell is designed based on the dense grid medium, as shown in Fig. 3a. It mainly consists of a cell strain detection pole, a cell dielectric layer, and a fixed cell plate, and the cell strain detection pole consists of several micro detection units connected by a bus line. Eventually, the electrical signal from the microdetection unit is collected and converted by the cell signal conversion circuit. The cell dielectric layer is made of a frothy silicon-lipid mixture. The fixed-cell plate design is based on rigid structures that prevent cutting orientation movement from affecting cell detection accuracy.

As shown in Fig. 3b, the smoke particles collide with the vertically oriented strain-inducing pole of the capacitive cell under the action of the blower. Under the effect of the collision force $F_{n}$, the dense grid medium will be compressed, which will change the distance between the fixed plate and the strain-inducing pole, thus changing the capacitance value of the capacitor. The detection of smoke particles is achieved by detecting the change in electrical signals caused by changes in capacitance.

Assuming that the invariant of the cell micro detection unit is $\Delta L$ after collision by particles, it can be expressed as follows:

2 $$\vartriangle L = \frac{{F_{n} d}}{{\rho_{A} EA_{S} }}$$

where $\rho_{A}$ is the filling rate of the cell dielectric layer, $E$ is the elastic recovery of the cell dielectric layer, $A_{S}$ is the area of the cell dielectric layer.

$F_{n}$ can be expressed as follows:

3 $$F_{n} = \delta_{i} F_{fan} R_{i}$$

where $\delta_{i}$ is the inertia coefficient of particle type $i$, $R_{i}$ is the diameter of particle type $i$, and $F_{fan}$ is the driving force of the blower to the particles. Furthermore, capacitance variations can be obtained after the cell micro detection unit collides with particles, as shown in Eq. (4), and the sensitivity can be expressed as Eq. (5).

4 $$C_{{\vartriangle L_{i} }} = \frac{{\varepsilon A_{i} }}{{d - \vartriangle L_{i} }} = \frac{{\varepsilon A_{i} }}{{d - \frac{{\delta_{i} F_{fan} R_{i} d}}{{\rho_{A} EA_{S} }}}}$$

5 $$\frac{{\partial C_{{\vartriangle L_{i} }} }}{{\partial R_{i} }} = \frac{{\varepsilon A_{i} \rho_{A} EA_{s} }}{{d\left( {\rho_{A} EA_{S} - \delta_{i} F_{fan} R_{i} d} \right)^{2} }}$$

where $A_{i}$ is the sensing electrode area of the micro detection unit and $\vartriangle L_{i}$ is the invariant of the cell micro detection unit after collision by particles. Because $F_{n}$ has a much lesser impact than $\rho_{A} EA_{s}$, the impact from $F_{n}$ can be ignored. At this point, the sensitivity can be expressed as

6 $$\frac{{\partial C_{{\vartriangle L_{i} }} }}{{\partial R_{i} }} \approx \frac{{\varepsilon A_{i} }}{{d\rho_{A} EA_{s} }}$$

A mixture of flexible body and gas gaps formed between the cell strain detection pole and the fixed cell plate. Equation (6) shows that the filling rate of the mixture on the cell dielectric layer should be reduced to improve the sensitivity.

## Signal output model and algorithm model

### Model of the output signal from the particle analysis structure

**Figure 4:**
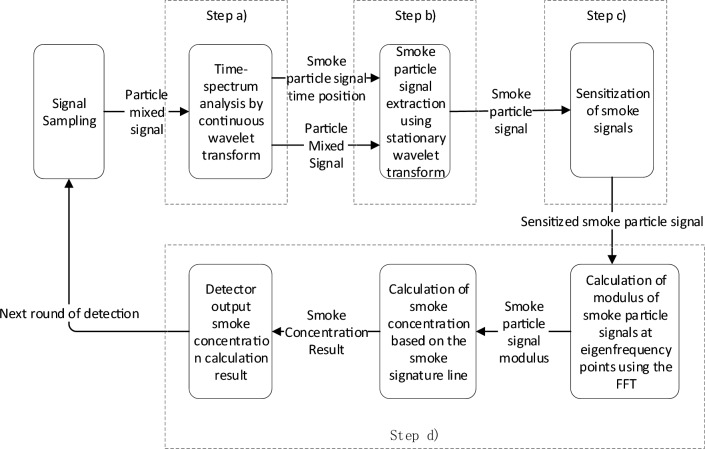
The flowchart of the multiscale smoke particle concentration detection algorithm.

The capacitance changes when the flexible capacitive plate is collided by particles. Because a fixed DC voltage is applied between two plates, an alternating current will produce a change in capacitance, the amplitude of which is the superposition of all weak AC signals caused by collisions between particles (including smoke particles and interfering particles) and capacitive cells, and the signal will be output by the signal stacker between two plates. The mathematical model has been developed by assuming sinusoidal currents.

7 $$I_{sum} = U_{0} \cdot \frac{{dC_{sum} }}{dt} = U_{0} \cdot \left[ {\frac{{d\left( {C_{{\vartriangle L_{1} }} } \right)}}{dt} + \frac{{d\left( {C_{{\vartriangle L_{2} }} } \right)}}{dt}} \right]$$

where $I_{sum}$ is the total alternating current signal synthesized by the signal stacker and, $U_{0}$ is the constant voltage between capacitor's terminal,$C_{sum}$ is the superposition of changes in the capacitance of the capacitor. The AC voltage signal is generated on the precision resistor in series between two signal stacks.

8 $$U_{sum} = I_{sum} *R_{samp}$$

where $R_{samp}$ is the electrical resistance of the precision sampling resistor, and it has a resistance value of 10MΩ, $U_{sum}$ is the AC voltage applied to the precision sampling resistor. A superposition of sinusoidal voltages with different frequencies and amplitudes will be formed after filtering and amplification by the signal processing circuit (as shown in Fig. 1).

9 $$U\left( t \right) = \sum\limits_{{R_{i} = R_{s} ,R_{{N_{1} }} ,R_{{N_{2} }} \cdots }} {A_{{R_{i} }} \cdot \sin \left[ {\omega_{{R_{i} }} \cdot t + \varphi } \right]}$$

where $R_{i}$ is the diameter of different particles, $R_{s}$ is the diameter of smoke particles to be detected, $R_{{N_{1} }}$, $R_{{N_{2} }}$, et al. are the diameters of interfe ring particles, $\omega_{{R_{i} }}$ is the frequency of the signal produced by particles with a diameter $R_{i}$, $A_{{R_{i} }}$ is the amplitude of the signal produced by particles with a diameter $R_{i}$, $\varphi$ is the offset angle of the signal, and $t$ is the time (Fig. 4).

### Smoke concentration detection algorithm

#### Overall design of the multiscale smoke particle concentration detection algorithm

The signal output of the detector is in this form the superposition of signals generated by particles at different times. The weak signal needs to be amplified with the signal enhancement technique because the size of the smoke particles is tiny. These drawbacks prevent the use of a single method for signal processing from meeting the demand for smoke concentration detection. The multiscale smoke concentration detection algorithm is a combinatorial algorithm for continuous wavelet transform, smooth wavelet transform, sensitization of smoke signals, and single-frequency point concentration calculations. Therefore, the multiscale smoke concentration detection algorithm—a combination of multiple signal analysis methods—will be used for this detection, and its main steps can be divided as follows:

- First, determine the time position of the smoke particle signal in the detector output signal.
- After that, the smoke particle signal needs to be extracted.
- Subsequently, the signal after extraction is sensitized and amplified.
- Finally, the smoke concentration is calculated via single-frequency analysis.

#### Time‒frequency analysis of signals

First, a time-spectrum analysis of the detector output signal is performed via a continuous wavelet transform along the time axis, and the moment at which the smoke particle signal appears is determined. The continuous wavelet transform of the continuous signal $f\left( t \right)$ can be expressed as follows:

10 $$WT_{f} \left( {a,b} \right) = \left\langle {f(t),\psi_{a,b} \left( t \right)} \right\rangle \frac{1}{\sqrt a }\int_{ - \infty }^{ + \infty } {f\left( t \right)} \psi^{*} \left( {\frac{t - b}{a}} \right)dt$$

where $a$ is the scale parameter of the wavelet function, $b$ is the translation parameter of the wavelet function, $\psi_{a,b} \left( t \right)$ is the wavelet basis function for parameters $a$ and $b$, $\psi^{*} \left( t \right)$ is the conjugate function of the wavelet basis function, and $f\left( t \right)$ is the source signal function.

The relationship between the wavelet decomposition scale and signal frequency after transformation can be expressed as follows:

11 $$f_{a} = \frac{{f_{c} f_{s} }}{a}$$

where $f_{a}$ is the actual signal frequency after decomposition, $f_{c}$ is the center frequency of the wavelet basis function, and $f_{s}$ is the sampling frequency of the signal. According to the sampling theorem, the value ranges of the scale parameter satisfy $a \in \left[ {2f_{s} ,\infty } \right]$ so that the value ranges of the frequency of the wavelet basis function can satisfy $f_{c} \in \left[ {0,f_{s} /2} \right]$.

#### Smoke particle signal separation

In addition, the smoke particle signal is extracted from the detector output signal by a stationary wavelet transform.

In the stationary wavelet transform, the scale parameter $a$ needs to be discretized, and the translation parameter $b$ remains constant so that the signal after the transform has the same length as the original signal $f\left( t \right)$. The stationary wavelet transform can be obtained through discrete sampling to the scale parameter $a$ within the binary sequence $\{ 2^{j} \}$ (where $j \in Z$).

12 $$SWT_{f} \left( {j,b} \right) = \left\langle {f\left( t \right),\psi_{a,b} \left( t \right)} \right\rangle = \frac{1}{{\sqrt {2^{j} } }}\int_{ - \infty }^{ + \infty } {f\left( t \right)} \psi^{*} \left( {\frac{t - b}{{2^{j} }}} \right)dt,j \in Z$$

Equation (12) shows that only the scale parameter $a$ is discretized by the stationary wavelet transform, and the translation parameter $b$ remains constant. In this way, the wavelet coefficients are all retained, and the length of the wavelet coefficients remains constant after each transform.

There are two ways of upsampling and downsampling at the same time so that the lengths of the signal between the original signal and the high- and low-frequency coefficients after the transform remain constant when the original signal is disintegrated by the stationary wavelet transform. This sampling mode is achieved by interpolating $2^{j}$ zeros between the two coefficients of the high-pass and low-pass filters; the high-pass and low-pass filter coefficients are stripped in this way; and the high-pass and low-pass filters in the transformation can be expressed as follows:

13 $$g\left( k \right) = \left\{ {\begin{array}{*{20}l} {g\left( {\frac{k}{{2^{j} }}} \right),} \hfill & {k = 2^{j} m} \hfill \\ {0,} \hfill & {others} \hfill \\ \end{array} } \right.$$

14 $$h\left( k \right) = \left\{ {\begin{array}{*{20}l} {h\left( {\frac{k}{{2^{j} }}} \right),} \hfill & {k = 2^{j} m} \hfill \\ {0,} \hfill & {others} \hfill \\ \end{array} } \right.$$

where $j,k,m \in Z$, $g\left( k \right)$ and $h\left( k \right)$ denote the unit response functions of the high-pass and low-pass filters, respectively.

Furthermore, the decomposition based on the Mallat algorithm can be obtained as follows:

15 $$\left\{ {\begin{array}{*{20}c} {S_{j + 1} \left( n \right) = \sum\limits_{k = 1}^{M} {S_{j} \left( k \right)g^{ * } \left( {k - 2n} \right)} } \\ {d_{j + 1} \left( n \right) = \sum\limits_{k = 1}^{M} {d_{j} \left( k \right)h^{ * } \left( {k - 2n} \right)} } \\ \end{array} ,\;j = 0,1, \cdots J} \right.$$

where $j$ is the decomposition depth of the Mallat algorithm, $J$ is the number of decompositions of the signal, $n$ is the degree of decomposition of the signal, $k$ is the order number of the decomposed sequence, $M$ is the sampling point upper limit of the decomposed sequence, and $S_{j} \left( k \right)$ and $d_{j} \left( k \right)$ denote the coefficients of the high-pass and low-pass filters, respectively, at the *j*th signal decomposition.

The detector output signal, which includes the smoke particle signal period, is decomposed by the stationary wavelet transform based on the Mallat algorithm. Suppose that the eigenfrequency of the awaiting detection smoke particle signal is $\omega_{{R_{S} }}$ and that the eigenfrequency of the interfering particle signal is $\omega_{{R_{i} }}$. The signal that contains only smoke particles can be acquired after $i$ the step of stationary wavelet decomposition.

**Figure 5:**
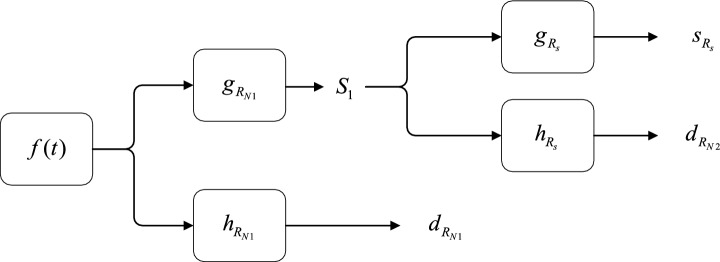
The signal decomposition diagram of the detector output signal by the stationary wavelet transform.

In Fig. 5, 2-s2-step decomposition is shown as an example. First, the original signal $f\left( t \right)$ is decomposed by high-pass and low-pass filters with coefficients $g_{{R_{{N_{1} }} }}$ and ${h}_{{R}_{N1}}$, respectively, and the signal $S_{1}$ filters the interference caused by interference particles of size $R_{{N_{1} }}$ and the interference signal $d_{{R_{{N_{1} }} }}$ generated by particles of this size. Subsequently, the signal $S_{1}$ is decomposed again by another high-pass and low-pass filter with coefficients ${g}_{{R}_{s}}$ and ${h}_{{R}_{s}}$, respectively, and the signal $S_{{R_{S} }}$ contains only the signal generated by smoke particles and the signal $d_{{R_{{N_{2} }} }}$ generated by interference particles of size $R_{{N_{1} }}$.

The relationship between the coefficients $g_{{R_{{N_{1} }} }}$ and $h_{{R_{{N_{1} }} }}$ of high-pass and low-pass filters in the first decomposition layer and the eigenfrequency $\omega_{{R_{{N_{1} }} }}$ of the interference signal caused by particles with size $R_{{N_{1} }}$ can be expressed as follows:

16 $$g_{{R_{{N_{1} }} }} = \beta_{{R_{{N_{1} }} }} \omega_{{R_{{N_{1} }} }} g\left( {\frac{{k_{{N_{1} }} }}{{2^{{j_{{N_{1} }} }} }}} \right)$$

17 $$h_{{R_{{N_{1} }} }} = \beta_{{R_{{N_{1} }} }} \omega_{{R_{{N_{1} }} }} h\left( {\frac{{k_{{N_{1} }} }}{{2^{{j_{{N_{1} }} }} }}} \right)$$

where $g\left( {\frac{{k_{{N_{1} }} }}{{2^{{j_{{N_{1} }} }} }}} \right)$ and $h\left( {\frac{{k_{{N_{1} }} }}{{2^{{j_{{N_{1} }} }} }}} \right)$ are the unit response functions of the high-pass and low-pass filter decomposition depths, respectively $N_{1}$, and $\beta_{{R_{{N_{1} }} }}$ is the correction coefficient for the eigenfrequency $\omega_{{R_{{N_{1} }} }}$.

Similarly, the relationship between the coefficients $g_{{R_{S} }}$ and $h_{{R_{S} }}$ of the high-pass and low-pass filters in the second decomposition layer and the eigenfrequency $\omega_{{R_{s} }}$ of the smoke signal caused by particles of size $R_{s}$ can be expressed as follows:

18 $$g_{{R_{S} }} = \beta_{{R_{S} }} \omega_{{R_{S} }} g\left( {\frac{{k_{{N_{S} }} }}{{2^{{j_{{N_{S} }} }} }}} \right)$$

19 $$h_{{R_{S} }} = \beta_{{R_{S} }} \omega_{{R_{S} }} h\left( {\frac{{k_{{N_{S} }} }}{{2^{{j_{{N_{S} }} }} }}} \right)$$

where $g\left( {\frac{{k_{{N_{S} }} }}{{2^{{j_{{N_{S} }} }} }}} \right)$ and $h\left( {\frac{{k_{{N_{S} }} }}{{2^{{j_{{N_{S} }} }} }}} \right)$ are the unit response functions of the high-pass and low-pass filter decomposition depths, $N_{S}$ respectively, and $\beta_{{R_{S} }}$ is the correction coefficient for the eigenfrequency $\omega_{{R_{s} }}$.

#### Signal sensitization and smoke concentration calculations

**Figure 6:**
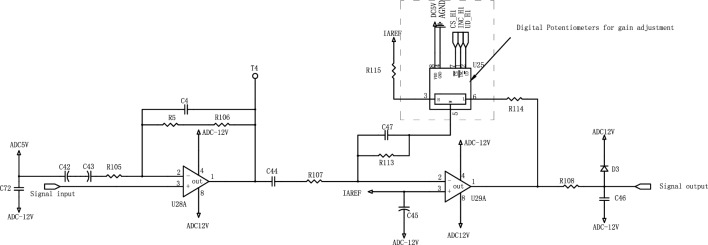
AC signal gain amplifier circuit schematic.

A programmable circuit, as shown in Fig. 6, is included in the signal processing circuit in Fig. 1. The circuit comprises 2 operational amplifiers (op. amps.) U28A and U29A, and a digital potentiometer U25. The very low-amplitude raw output at the sensitive element is amplified through a two-stage amplifier circuit consisting of U28A and U29A. The gain of the output signal can be adjusted by changing the tap position of the digital potentiometer U25. Finally, the processed analog signal is passed to an analog-to-digital converter (ADC). "ADC5V" is the DC 5 V power supply for the analog simulation circuit section. Capacitor C46 is used to filter the signal. Diode D3 is used to provide voltage-limited protection for the ADC signal. When the ADC voltage exceeds 12 V, diode D3 conducts to limit the ADC signal to 12 V. Electrolytic capacitors C42 and C43 are connected in reverse series to form an unpolarized capacitor of halved capacitance. This is to double the voltage rating of the capacitor.

Where $S_{{R_{S} }}^{*}$ is the sensitized smoke particle concentration signal and $Gain$ is the signal magnification.

The fast Fourier transform (FFT) algorithm was used to calculate the modulus of a single frequency point after separation and sensitization. Near the characteristic frequency ω of the smoke particle signal, the characteristic frequency modulus $M_{{R_{S} }}$ can be obtained.

Finally, the smoke concentration can be calculated by bringing the modulus $M_{{R_{S} }}$ into the smoke concentration characterization line as follows:

20 $$Col_{{R_{S} }} = \gamma_{{R_{S} }} \times M_{{R_{S} }} + \rho_{{R_{S} }}$$

where $Col_{{R_{S} }}$ is the calculated smoke concentration, $\gamma_{{R_{S} }}$ is the slope of the smoke concentration characteristic line, and $\rho_{{R_{S} }}$ is the constant of the smoke concentration characteristic line.

## Experimental

### Introduction of the experimental device

**Figure 7:**
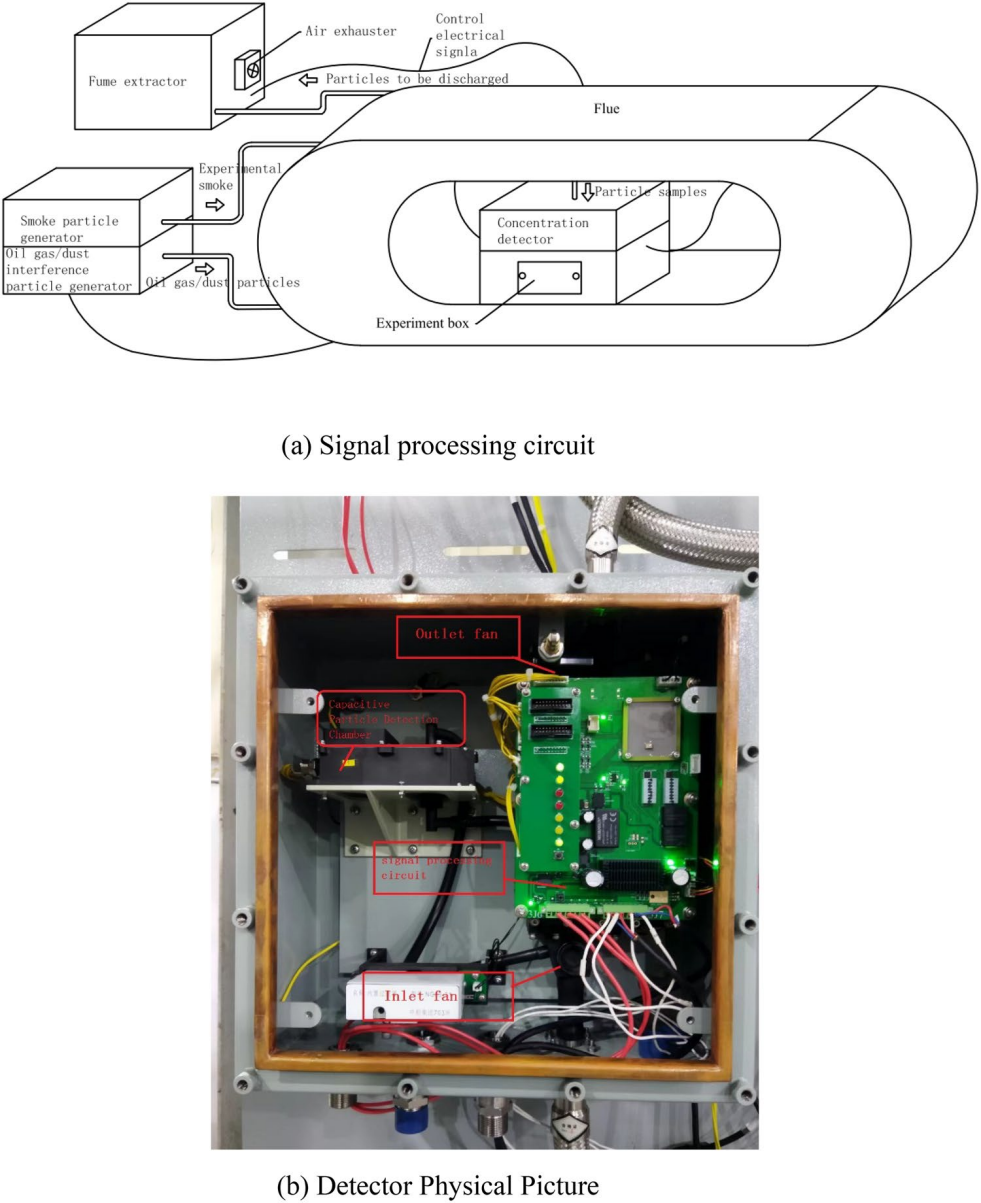
Smoke concentration experimental device structure diagram and detector physical picture.

A smoke concentration experimental device was used to test the performance of this detector, as shown in Fig. 7a. The experiment box is the chamber that place the detector used to experiment on it. The experimental equipment is produced by Beijing Yuanhengliye Corporation, and its model number is SMK-2000. This experimental device is composed of a smoke particle generator, an interference generator, a concentration detection device, an experiment box, etc.etc. The smoke particle generator generates simulated smoke particles at different concentrations during a fire. An interference generator generates oil gas or dust particles of different sizes and concentrations in different environments. The flue mixture of the above particles was generated, and uniform particles were mixed into the experimental box when the concentration detected by the concentration detection device reached the set conditions. The concentration of various particles produced by the experimental device is measured by a sophisticated optical densitometer inside the device. The device regulates the particle concentration based on the feedback signal. As a result, the concentration accuracy of various particles generated in this device is 0.0001 PPM.

### Limit concentration detection experiment

**Figure 8:**
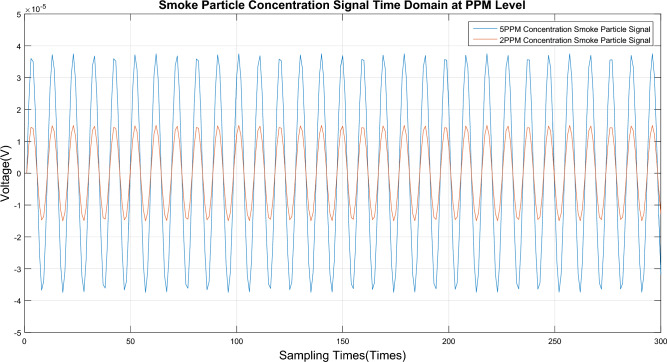
Time domain signal for diagram limit concentration detection.

**Figure 9:**
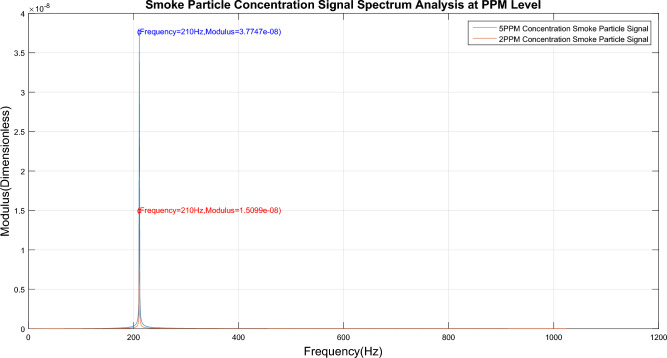
Limit concentration detection spectrum.

The smoke particles were separated at concentrations of 2.0 ppm and 5.0 ppm by this device, after which these particles were used to conduct a concentration limit detection experiment on the detector. The time domain signal of the smoke particle output from the detector is shown in Fig. 8, and its spectrum is shown in Fig. 9. The eigenfrequency $\omega_{{R_{s} }}$ of smoke particles can be determined to be 210 Hz.

The specific calculations are shown in Table 1, and the deviations are expressed in parts-per-million (PPM) scale. The deviation is the difference between the concentration (the value shown on the concentration meter on the test set) produced by the device (shown in Fig. 7) and the actual concentration (the concentration is calculated by taking the modulus calculated by the detector at the smoke particle characteristic frequency point $\omega_{{R_{s} }}$ into Eq. (20)) measured by the detector.

**Table 1:** Smoke concentration experiment results.

Smoke concentration (PPM)	Modulus (dimensionless)	Detection concentration (PPM)	Deviation (PPM)
2	0.000150994058	2.3	0.2
5	0.0003774835145	5.2	0.3

As shown in Table 1, the results are 5.2PPM and 2.3PPM, with a detection deviation of less than 0.5PPM when the detector detects smoke particles at concentrations of 2PPM and 5PPM, respectively.

### Smoke concentration detection experiment

**Figure 10:**
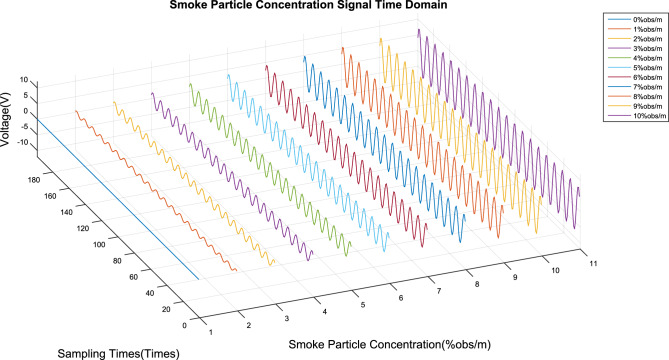
Time domain signal of 0–10% obs/m smoke particle concentration.

**Figure 11:**
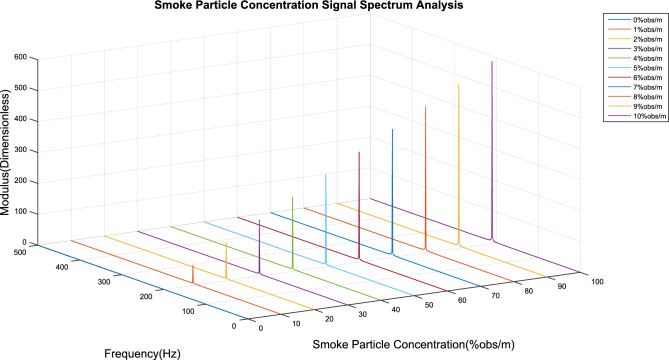
Spectrum of 0–10% obs/m smoke particle concentration.

Smoke particles with concentrations ranging from 0% obs/m to 10% obs/m were separated by this device, and these particles were used to conduct a concentration limit detection experiment on the detector. The time domain and signal spectrum are shown in Figs. 10 and 11, respectively, and the detection results are shown in Table 2.

**Table 2:** Smoke concentration experiment results.

Smoke concentration (%obs/m)	Modulus (dimensionless)	Detection concentration (%obs/m)	Deviation (PPM)
1	58.2667029	1.0000003	0.3
2	116.5334059	2.0000002	0.2
3	174.8001084	3.0000003	0.3
4	233.0668105	4.0000004	0.4
5	291.3335132	5.0000003	0.3
6	349.6002158	6.0000002	0.2
7	407.8669182	7.0000003	0.3
8	466.1336209	8.0000004	0.4
9	524.4003241	9.0000002	0.2
10	582.6670265	10.0000003	0.2

### Anti-interference ability experiment

Mixed particles with 6% obs/m oil gas particles, 7% obs/m large dust interference particles, 8% obs/m small dust interference particles, and 2% obs/m smoke particles were prepared, and mixed particles were pumped into the experimental box of this device for an anti-interference experiment.

**Figure 12:**
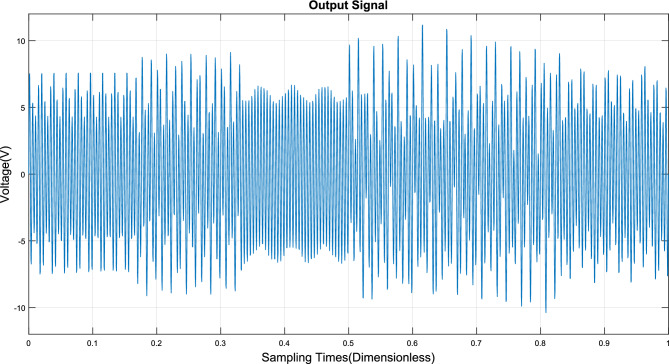
Interference experiment detector signal output diagram.

**Figure 13:**
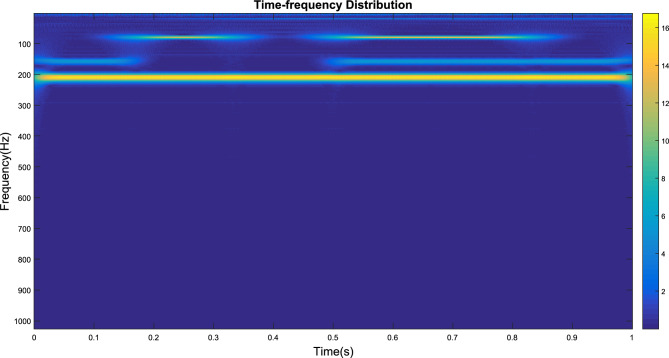
Time‒frequency distribution.

The signal output from this detector is shown in Fig. 12. Subsequently, the signal of mixing with various particles is transformed by a continuous wavelet transform to obtain the time–frequency distribution, as shown in Fig. 13. From that figure, it can be seen that there are 4 main frequencies, and the signal with a frequency of 200 Hz is distributed over the whole timeline.

**Figure 14:**
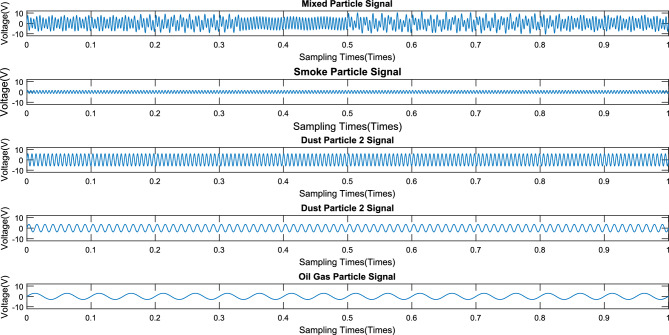
Signal decomposition diagram.

**Figure 15:**
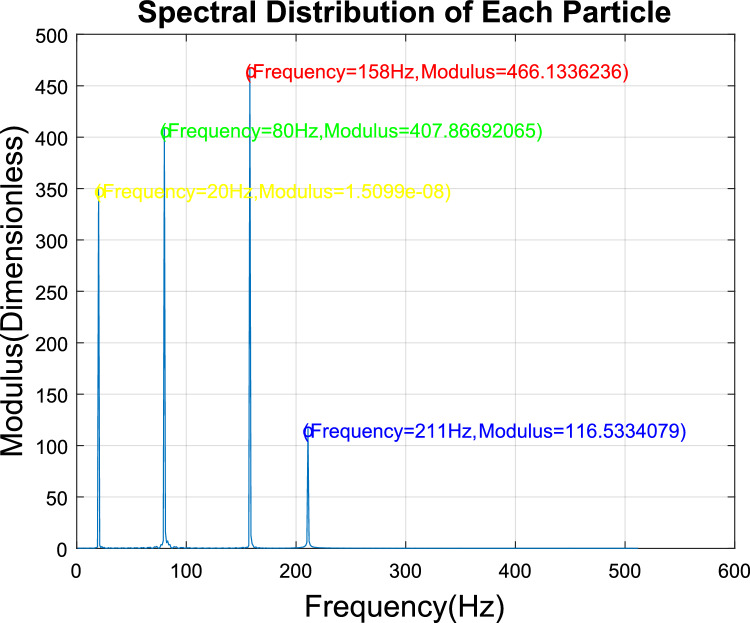
Spectral distribution of each particle.

Furthermore, the signals generated by mixed particles are decomposed to obtain the smoke particle signal. The time domain diagrams before and after signal decomposition are shown in Fig. 14. Then, a spectral analysis of the various particle signals after decomposition was performed, as shown in Fig. 15. It is apparent from this figure that there are 4 main frequency points at 20 Hz (oil gas particle signal), 80 Hz (large dust interference particle signal), 158 Hz (small dust interference particle signal), and 210 Hz (smoke particle signal).

Finally, the smoke particle concentration was calculated, and the results are shown in Table 3. The detection concentration was 2.0000007% obs/m, and the detection accuracy was higher than that of the PPM.

**Table 3:** Anti-interference ability experiment results.

Smoke concentration (%obs/m)	Modulus (dimensionless)	Detection concentration (%obs/m)	Deviation (PPM)
2	116.5334079	2.0000007	0.7

From the experimental results, it can be seen that the detector separates the smoke particle signals. Therefore, it can distinguish between smoke particles and other interfering particles. The detector has excellent false alarm resistance and reliability.

### Comparison with previously reported methods

Compare the performance of the capacitive particle analysis smoke detector with other mainstream detectors in the industry, as shown in Table 4. The other three sensors are %obs/m level, which is significantly less accurate than the PPM level reached by the capacitive particle analysis smoke detector. The capacitive particle analysis smoke detector has a sensitivity level of PPM, which is significantly higher than the other three detectors. Only the capacitive particle analysis smoke detector can be able to recognize the different particle types. Therefore, it has higher anti-false alarm capability and reliability.

**Table 4:** Comparison of detector performance.

Model	Manufacturer	Accuracy	Sensitivity	The ability to recognize different particle types
JTY-GD-TC9800	VESDA(UAS)	1%obs/m	0.5% obs/m	Not possess
U5014A4N13F	DET-TRONICS (UAS)	3%-5% obs/m	0.5% obs/m	Not possess
JTY-BK721	BOKANG (China)	3%-5% obs/m	1% obs/m	Not possess
The capacitive particle analysis smoke detector	HIT	2.0PPM	0.2PPM	Possess

## Conclusions

1. The limit of the smoke particle concentration measured by the detector reaches the PPM. The designed capacitive detection cell effectively improves the sensitivity of the detector and can measure the concentration of smoke particles effectively at the PPM level.
2. The designed detector can effectively detect smoke particles at a concentration of 0–10% obs/m, and the detection accuracy can be higher than that of the PPM. The newly designed capacitive particle analysis detector and multiscale smoke particle concentration detection algorithm can perform high-precision detection of smoke particles at various concentrations.
3. Even when there is interference from oil, gas, or dust particles, the detector can still accurately detect at a higher level than the PPM. This paper shows that capacitive particle analysis and detection structures based on capacitive detection cells combined with a multiscale smoke particle concentration detection algorithm can effectively improve the reliability of detectors to eliminate the influence of other interfering particles on detector performance in complex environments.

## Data Availability

All data generated or analysed during this study are included in this published article.
